# Supplementary material for: Combination of FDG PET/CT Radiomics and Clinical Parameters for Outcome Prediction in Patients with Hodgkin’s Lymphoma
Source: Cancers (Basel). 2023 Mar 30;15(7):2056. doi: 10.3390/cancers15072056 (PMC10093084; doi:10.3390/cancers15072056)
Supplement: Supplementary file 1 [file cancers-15-02056-s001.zip › cancers-2209373-supplementary.pdf]

Supplemental Table: UVA results for clinical variables.

Table S1. Effect on PFS

Covariate	HR(95%CI)	p-value	Global p-value
**GENDER**			0.27
Female	Reference		
Male	2.15 (0.56,8.3)		
**PATH SUBTYPE TEXT**			0.84
Classical Hodgkin's lymphoma, NOS	Reference		
Mixed cellularity classical Hodgkin lymphoma (MCHL)	7e-08 (0e+00,Inf)	1	
Nodular lymphocyte predominant Hodgkin lymphoma (NLPHL)	1.39 (0.09,22.26)	0.81	
Nodular sclerosis classical Hodgkin lymphoma (NSHL)	2.36 (0.29,18.85)	0.42	
**BULK SIZE**	1.25 (1.08,1.46)		**0.0035**
**Overall stage**			0.13
I + II	Reference		
III + IV	2.85 (0.74,11.01)		
**BSYMPTOMS**			**0.025**
no	Reference		
yes	0.24 (0.07,0.84)		
**REGIMEN NAME CHEMO**			0.53
ABVE-PC	Reference		
CT-A-AVD	1.3e-08 (0e+00,Inf)	1	
CT-A+AVD	0.48 (0.03,7.73)	0.61	
LY-ABVD	0.18 (0.02,1.45)	0.11	
OEPA/COPDAC	1.3e-08 (0e+00,Inf)	1	
**CHEMOTHERAPY COMPLETED AS PLANNED**			**0.0062**
No	Reference		
Yes	0.11 (0.02,0.54)		
**RESPONSE TO CHEMO**			**<0.001**
	Reference		
CR - Completer response	1 (2.4e-95,4.2e+94)	1	
PD - Progressive disease	1.9e+04 (4.9e+03,7.1e+04)	**<0.001**	
PR - Partial response	6.5e+03 (1.6e+03,2.7e+04)	**<0.001**	
SD - Stable disease	1.4e+05 (1.1e+04,1.7e+06)	**<0.001**	
**Hemoglobin RESULT**			
Normal	Reference		**0.01**
Abnormal	6.3e+08 (0e+00,Inf)		1
**WBC RESULT**			
Abnormal	Reference		0.27

Normal	0.38 (0.11,1.33)	0.13
**Neutrophils RESULT**		
Abnormal	Reference	0.22
Normal	0.17 (0.04,0.8)	**0.025**
**Lymphocites RESULT**		
Abnormal	Reference	0.19
Normal	0.26 (0.05,1.21)	0.085
**Eosinophils RESULT**		
Abnormal	Reference	0.73
Normal	0.6 (0.17,2.12)	0.43
**ESR RESULT**		
Abnormal	Reference	**<0.001**
Normal	3.3e-09 (0e+00,Inf)	1
**LDH RESULT**		
Abnormal	Reference	0.47
Normal	0.61 (0.16,2.37)	0.48
**ALP RESULT**		
Normal	Reference	**0.035**
Abnormal	11.73 (3.29,41.81)	**<0.001**
**ALT RESULT**		
Abnormal	Reference	0.56
Normal	8.7e+07 (0e+00,Inf)	1
**AST RESULT**		
Abnormal	Reference	0.25
Normal	7.6e+07 (0e+00,Inf)	1
**Albumin RESULT**		
Normal	Reference	**<0.001**
Abnormal	11.54 (2.39,55.67)	**0.0023**
**Creatinine RESULT**		
Abnormal	Reference	0.5
Normal	2.6e+07 (0e+00,Inf)	1
**Calcium RESULT**		
Abnormal	Reference	0.071
Normal	0.72 (0.09,5.68)	0.76

Table S2. Effect on Radiotherapy outcome.

**Covariate**	**OR(95%CI)**	**p-value**	**Global p-value**
**GENDER**			0.82
Female	Reference		
Male	1.1 (0.48,2.54)		
**PATH SUBTYPE TEXT**			0.4
Classical Hodgkin's lymphoma, NOS	Reference		
Mixed cellularity classical Hodgkin lymphoma (MCHL)	3.11 (0.28,34.42)	0.35	

Nodular lymphocyte predominant Hodgkin lymphoma (NLPHL)	0.93 (0.2,4.37)	0.93	
Nodular sclerosis classical Hodgkin lymphoma (NSHL)	0.58 (0.19,1.79)	0.35	
**BULK SIZE**	0.99 (0.88,1.12)		0.88
**Overall stage**			**<0.001**
I + II	Reference		
III + IV	0.05 (0.02,0.14)		
**BSYMPTOMS**			**0.0048**
no	Reference		
yes	4.51 (1.58,12.83)		
**COMPLETED AS PLANNED CHEMO**			0.36
No	Reference		
Yes	2.93 (0.29,29.32)		
**RESPONSE TO CHEMO 1**			0.81
	Reference		
CR - Completer response	2.7e-08 (0e+00,Inf)	1	
PD - Progressive disease	2.7e-08 (0e+00,Inf)	0.99	
PR - Partial response	1.55 (0.32,7.58)	0.59	
SD - Stable disease	2.7e-08 (0e+00,Inf)	1	
**Hemoglobin RESULT**			**0.0012**
Normal	Reference		
Abnormal	0.23 (0.09,0.56)		
**WBC RESULT**			0.39
Abnormal	Reference		
Normal	1.46 (0.61,3.5)		
**Neutrophils RESULT**			0.42
Abnormal	Reference		
Normal	1.42 (0.6,3.34)		
**Lymphocites RESULT**			**0.0086**
Abnormal	Reference		
Normal	3.25 (1.35,7.82)		
**Eosinophils RESULT**			0.61
Abnormal	Reference		
Normal	1.27 (0.5,3.24)		
**ESR RESULT**			**0.028**
Abnormal	Reference		
Normal	3.6 (1.15,11.29)		
**LDH RESULT**			0.22
Abnormal	Reference		
Normal	1.73 (0.72,4.13)		
**ALP RESULT**			**0.031**
Normal	Reference		
Abnormal	0.18 (0.04,0.85)		
**ALT RESULT**			0.11
Abnormal	Reference		

Normal	0.36 (0.1,1.27)	
**AST RESULT**		0.6
Abnormal	Reference	
Normal	0.66 (0.14,3.14)	
**Albumin RESULT**		0.18
Normal	Reference	
Abnormal	0.48 (0.17,1.4)	
**Creatinine RESULT**		0.94
Abnormal	Reference	
Normal	0.93 (0.13,6.92)	
**Calcium RESULT**		0.9
Abnormal	Reference	
Normal	0.9 (0.17,4.75)	
